# Metabolic cost and mechanical work of walking in a virtual reality emulator

**DOI:** 10.1007/s00421-023-05306-0

**Published:** 2023-09-30

**Authors:** Francesco Luciano, Alberto E. Minetti, Gaspare Pavei

**Affiliations:** Locomotion Physiomechanics Laboratory, Department of Pathophysiology and Transplantation – Division of Physiology, University of Milan, Via Mangiagalli 32, 20133 Milan, Italy

**Keywords:** Metabolic power, Bioenergetics, Omnidirectional treadmill, Active video gaming, Biomechanics

## Abstract

**Purpose:**

The purpose of this study was to investigate the metabolic cost (C), mechanical work, and kinematics of walking on a multidirectional treadmill designed for locomotion in virtual reality.

**Methods:**

Ten participants (5 females, body mass 67.2 ± 8.1 kg, height 1.71 ± 0.07 m, age 23.6 ± 1.9 years, mean ± SD) walked on a Virtuix Omni multidirectional treadmill at four imposed stride frequencies: 0.70, 0.85, 1.00, and 1.15 Hz. A portable metabolic system measured oxygen uptake, enabling calculation of C and the metabolic equivalent of task (MET). Gait kinematics and external, internal, and total mechanical work (W_TOT_) were calculated by an optoelectronic system. Efficiency was calculated either as W_TOT_/C or by summing W_TOT_ to the work against sliding frictions. Results were compared with normal walking, running, and skipping.

**Results:**

C was higher for walking on the multidirectional treadmill than for normal walking, running, and skipping, and decreased with speed (best-fit equation: C = 20.2–27.5·speed + 15.8·speed^2^); the average MET was 4.6 ± 1.4. Mechanical work was higher at lower speeds, but similar to that of normal walking at higher speeds, with lower pendular energy recovery and efficiency; differences in efficiency were explained by the additional work against sliding frictions. At paired speeds, participants showed a more forward-leaned trunk and higher ankle dorsiflexion, stride frequency, and duty factor than normal walking.

**Conclusion:**

Walking on a multidirectional treadmill requires a higher metabolic cost and different mechanical work and kinematics than normal walking. This raises questions on its use for gait rehabilitation but highlights its potential for high-intensity exercise and physical activity promotion.

**Supplementary Information:**

The online version contains supplementary material available at 10.1007/s00421-023-05306-0.

## Introduction

Promoting physical activity is critical for preventing and managing chronic diseases and is a focus of major global health initiatives (World Health Organization 2018, 2020; Bull et al. 2020). Virtual reality emulators offer a novel approach to this effort, as they can be combined with a multidirectional treadmill to allow people to walk in a virtual environment while playing active video games or receiving live feedback (Gao et al. 2015; Dębska et al. 2019; Polechoński et al. 2020). In these devices, users glide with their feet on a smooth surface while a harness keeps their body in place, allowing them to freely change their direction and speed. As the users can move freely in a safe manner and receive visual and auditory feedback, multidirectional treadmills have also been suggested as a rehabilitation tool for people with gait disorders (Soni and Lamontagne 2020; Janeh and Steinicke 2021).

Little is known about the energy demands of walking on multidirectional treadmills, but several factors suggest that they may differ from those of normal walking. The harness and platform restrict the users’ movement, which may impair their ability to move their body center of mass (BCoM) in a pendulum-like manner and increase the mechanical work required to raise and accelerate the BCoM (Cavagna et al. 1963, 1976, 1977). However, they may also provide support for body weight during walking, reducing the work done against gravity and hence the metabolic demands (Pavei et al. 2015). Data from Soni and colleagues also show that people walk with a higher stride frequency, shorter swing time, and longer stance time on multidirectional treadmills compared to normal walking (Soni and Lamontagne 2020), which can increase the work done to swing the limbs relative to the BCoM (Fenn 1930; Cavagna et al. 1977; Peyré-Tartaruga et al. 2021). In addition, people adopt a more ‘crouched’ posture on multidirectional treadmills, with higher knee and hip flexion and ankle dorsiflexion during the stance phase (Jochymczyk-Woźniak et al. 2019; Soni and Lamontagne 2020), and lower limb muscle activity (Soni and Lamontagne 2020), which may further increase the cost of walking (Pincheira et al. 2017). Finally, sliding the feet on the treadmill may require additional work to overcome friction along the walking direction.

Determining the energy requirements of walking on multidirectional treadmills would help tailoring their use in preventive medicine and rehabilitation. The World Health Organization recommends that children and adolescents should engage in an average of at least 60 daily minutes of moderate-to vigorous-intensity physical activity—i.e., one which increases the resting metabolic rate by at least threefold—and adults should engage in at least 150 min of moderate or 75 min of vigorous physical activity per week (World Health Organization 2020; Bull et al. 2020). While normal walking is generally a light-to-moderate physical activity, walking on a multidirectional treadmill may be more intense and help meet these recommendations. However, if the metabolic cost of walking on multidirectional treadmills is too high, it may limit the ability to maintain a given walking speed for an extended period of time (Wilkie 1980; di Prampero 1986; Morton 2006), which may limit its use in gait rehabilitation for individuals with reduced aerobic fitness due to advanced age (Fitzgerald et al. 1997; Schneider 2013), disease (Sietsema et al. 2020), or immobilization (Ried-Larsen et al. 2017), while making it suitable for high-intensity interval training (MacInnis and Gibala 2017). Therefore, the aim of the present study was to evaluate the metabolic demands and mechanical work of walking on a multidirectional treadmill used for locomotion in virtual reality.

## Materials and methods

### Participants

Ten healthy subjects (5 females, 5 males; body mass 67.2 ± 8.1 kg, height 1.71 ± 0.07 m, age 23.6 ± 1.9 years, mean ± standard deviation) were recruited. Participants were excluded if they reported cardiovascular, neurological, or musculoskeletal conditions that might affect gait. The local ethical committee approved the experimental protocol, and all subjects gave their written informed consent before the start of the study after becoming aware of the potential risks involved in the experimental sessions.

### Virtuix Omni®

**Fig. 1:**
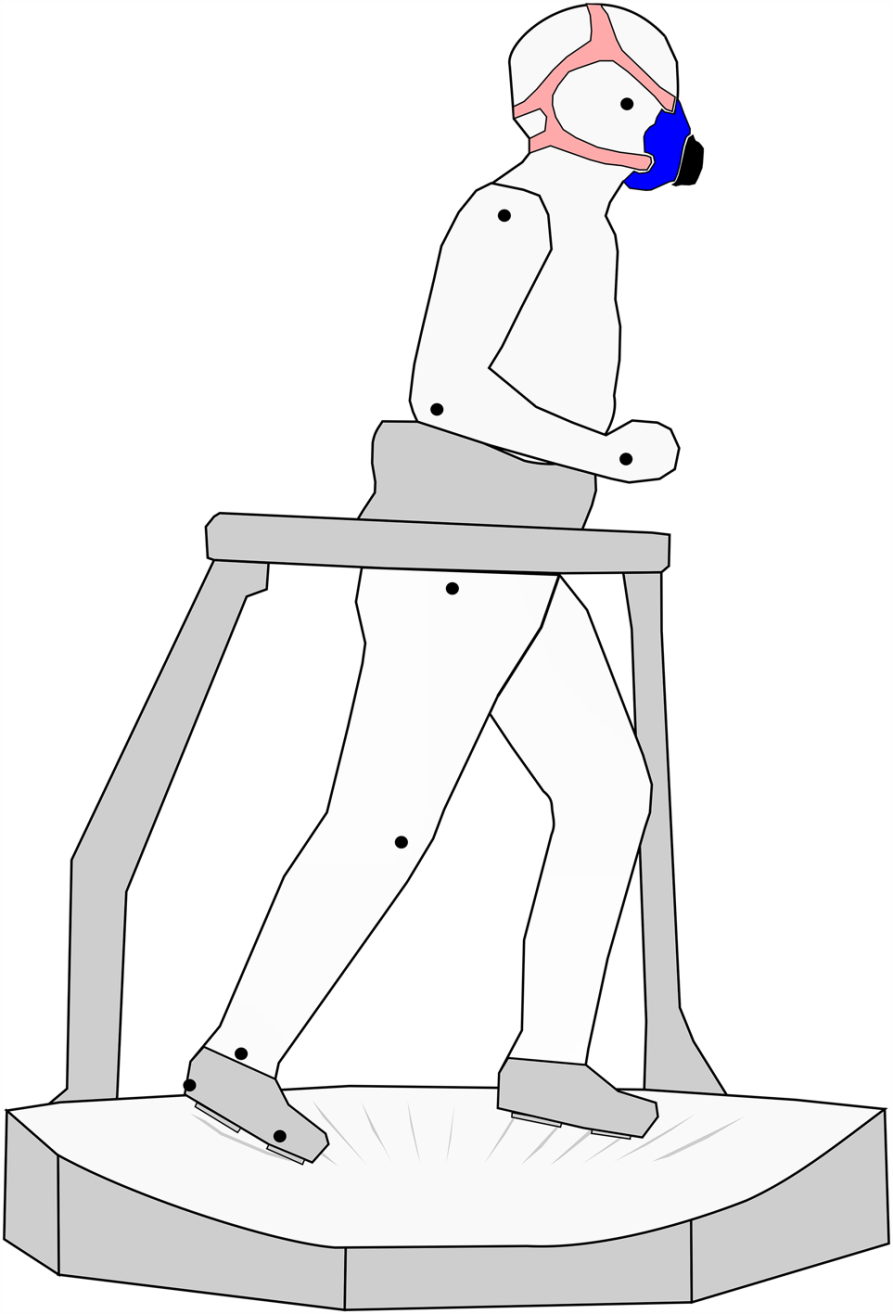
Experimental setup. The Virtuix Omni omnidirectional treadmill consists of a concave platform and a harness that constrains the user’s position while allowing rotation on the transverse and sagittal planes. The user’s feet slide on the concave surface with the help of special low-friction shoes. The black dots on one side of the participant indicate the position of the reflective markers used for this study

Virtuix Omni is a multidirectional treadmill designed for virtual reality gaming. The device consists of a concave platform on which the feet can slide, and a harness that keeps the participant centered with respect to the platform (Fig. 1; Supplementary Video 1). The feet can slide on the platform using special shoes with smooth sliding soles. The harness allows players to lean the trunk on the transverse and sagittal planes. The shape of the platform was characterized with a Vicon optoelectronic system and 17 reflective markers placed on a 1.01 m diameter: a second-order polynomial in the form *y* = 0.2694*x*^2^−0.0079*x*−0.0016 fitted the platform concavity with a coefficient of determination of 0.99, providing a reference threshold for determining gait events.

### Experimental procedure

Participants walked on the Omni multidirectional treadmill at four different stride frequencies (0.70, 0.85, 1.00 and 1.15 Hz) imposed by a metronome; such frequencies correspond to those that would elicit speeds between 0.28 and 1.94 m s^−1^ during normal walking (e.g., Saibene and Minetti 2003; Pavei et al. 2015). Metabolic cost, mechanical work, spatiotemporal parameters of gait, and sagittal plane kinematics were recorded during walking on the Omni and compared to those of 13 participants from Pavei et al (2015) who walked on a 'traditional' unidirectional treadmill (Ergo LG Woodway, USA).

### Familiarization

Each participant underwent two familiarization sessions before data collection. In the first one, participants became accustomed to walking on the Omni and tried all the stride frequencies used in the study. In the second session, they tried all four different stride frequencies again and were also asked to walk continuously for at least 4 min at 0.85 Hz and 1.00 Hz. Participants were free to choose their posture on the Omni, but were instructed not to lean on the harness with their upper limbs.

### Kinematics

The length of each stride was calculated as

1 $$\sum_{t={t}_{\mathrm{LHS}0}}^{{t}_{\mathrm{LTO}}}\sqrt{{{(x}_{{\mathrm{LHee}}_{\mathrm{i}}}-{x}_{{\mathrm{LHee}}_{\mathrm{i}-1}})}^{2}+ {{(y}_{{\mathrm{LHee}}_{\mathrm{i}}}-{y}_{{\mathrm{LHee}}_{\mathrm{i}-1}})}^{2}+ {{(z}_{{\mathrm{LHee}}_{\mathrm{i}}}-{z}_{{\mathrm{LHee}}_{\mathrm{i}-1}})}^{2} }+ \sum_{t={t}_{\mathrm{LTO}}}^{{t}_{\mathrm{LHS}1}}\sqrt{{{(x}_{{\mathrm{RHee}}_{\mathrm{i}}}-{x}_{{\mathrm{RHee}}_{\mathrm{i}-1}})}^{2}+ {{(y}_{{\mathrm{RHee}}_{\mathrm{i}}}-{y}_{{\mathrm{RHee}}_{\mathrm{i}-1}})}^{2}+ {{(z}_{{\mathrm{RHee}}_{\mathrm{i}}}-{z}_{{\mathrm{RHee}}_{\mathrm{i}-1}})}^{2},}$$

where t_LHS0_, t_LTO_ and t_LHS1_ are the times of consecutive left heel strikes, left toe-off and left heel strikes, respectively. The average progression speed was calculated as the product of stride length and stride frequency, and duty factor as the percentage of the stride period during which a foot was in contact with the ground; double contact time was also calculated. Sagittal projections of trunk, hip, knee, and ankle joint angles were reported and compared with those of normal walking; for this purpose, observations were grouped based on the measured walking speed (0.28 ± 0.14 m s^−1^ and 0.56 ± 0.14 m s^−1^).

### External mechanical work, energy recover,y and internal mechanical work

Three-dimensional body motion was collected by an eight-camera system (6 Vicon MX 1.3, 2 T20-S, Oxford Metrics, UK) sampling at 100 Hz the spatial coordinates of 18 reflective markers located on the main joint centers (Pavei et al. 2017). Marker positions were filtered through a ‘zerolag’ second-order Butterworth low-pass filter with a cutoff frequency detected by a residual analysis on each marker’s coordinate (Winter 1979). Acquisitions lasted 1 min and the time course of the 3D BCoM position was computed from an 11-segment model (Minetti et al. 1993; Pavei et al. 2017) based on Dempster inertial parameters of body segments (Winter 1979). From the BCoM 3D trajectory, the time course of potential (PE) and kinetic (KE) energies was computed to obtain the total mechanical energy (TE = PE + KE). The summation of all increases in TE time course constitutes the work done to accelerate and lift the BCoM (external mechanical work; Cavagna et al. 1963; Minetti et al. 1993), which was here mass and distance normalized (W_EXT_, J kg^−1^ m^−1^). Energy recovery, which measures the ability of the moving system to save energy in a pendulum-like way, was calculated according to Cavagna et al. (1976). The mass-specific and distance-normalized work necessary to rotate and accelerate limbs with respect to BCoM (internal kinetic mechanical work; W_INT,k_, J kg^−1^ m^−1^; Fenn 1930; Cavagna and Kaneko 1977; Minetti et al. 1993) was also calculated. All data were analyzed with purposely written Labview programs (release 10, National Instruments). After inspection of stereophotogrammetric data, mechanical variables were excluded for five acquisitions in which the harness for the multidirectional treadmill affected the quality of the marker data (Supplementary Data 1).

### Work done against sliding friction

The energy dissipated due to sliding friction between the shoes and the platform was estimated as follows. First, the static (*μ*_s_) and dynamic (*μ*_d_) sliding friction coefficients between the shoes and platform were measured to be 0.19 and 0.11, respectively, by means of platform tilting experiments (Hu et al. 2009; Supplementary Methods S1). Sliding friction is given by the product of the friction coefficient and the normal force on the sliding surface *F*_N_. During each stride, the work done against the average sliding friction force is hence given by $\overline{{F }_{N}} {\mu }_{d}d$, where *d* is the displacement of the body with respect to the platform and $\overline{{F }_{N}}$ is the average normal force. The mass- and distance-normalized mechanical work done against sliding against friction (J kg^−1^ m^−1^) is hence given by:

2 $${W}_{\mathrm{slide}}=\frac{\overline{{F }_{N}}}{m}{\mu }_{d}.$$

Assuming that over a walking stride $\overline{{F }_{N}}$ is equal to body weight, with a gravity acceleration g = 9.81 m s^−2^:

3 $${W}_{\mathrm{slide}}=g{\mu }_{d}\approx 1.1 \,J k{g}^{-1}{m}^{-1}.$$

### Total mechanical work

Total mechanical work (W_TOT_, J kg^−1^ m^−1^) was calculated in two ways. First, as classically defined (Cavagna and Kaneko 1977):

4 $${W}_{\mathrm{TOT}}={W}_{\mathrm{EXT}}+{W}_{\mathrm{INT},\mathrm{K}}.$$

Alternatively, a total mechanical work that also comprises the additional work done against sliding friction was calculated as:

5 $${W}_{\mathrm{TOT},\mathrm{WS}}={W}_{\mathrm{EXT}}+{W}_{\mathrm{INT},\mathrm{K}}+{W}_{\mathrm{slide}}.$$

### Metabolic cost

Pulmonary ventilation, oxygen consumption, and carbon dioxide production were measured breath by breath by a portable metabolic system, together with heart rate (K5, Cosmed, Italy). Prior to the experimental session, resting oxygen consumption ($\dot{V}{\text{O}}_{2}$, mlO_2_ kg^−1^ min^−1^) was measured while standing for 4 min. Participants then started walking on the Omni; each data acquisition lasted 4 min to reach a steady-state $\dot{V}{\text{O}}_{{2}}$. The metabolic cost of walking (C; J kg^−1^ m^−1^) (Margaria 1938; Schmidt-Nielsen 1972) was calculated as:

6 $$C=\frac{\left(\dot{V}{O}_{2\mathrm{ss}} - \dot{V}{O}_{2\mathrm{rest}}\right) Eq{O}_{2}}{v},$$

where $\dot{V}{\text{O}}_{{2ss}}$ and $\dot{V}{\text{O}}_{{2rest}}$ are the oxygen consumption during the last minute of walking and standing rest, respectively, *v* is the average progression speed (m s^−1^) obtained through kinematic analysis of the heel and metatarsus markers (see above), and *EqO*_*2*_ is the number of Joules released from the oxidative combustion of 1 ml of oxygen at a given respiratory exchange ratio (Lusk 1924). The metabolic equivalent of task (MET) was calculated as the ratio of $\dot{V}{\text{O}}_{{2ss}}$ and $\dot{V}{\text{O}}_{{2rest}}$ (Ainsworth et al. 1993). In all the conditions, exercise was at submaximal intensity (respiratory exchange ratio < 1). Participants were instructed to avoid strenuous exercise 24 h previously and avoid caffeine or food for at least 3 h before the experiment. Gait mechanics and energetics were assessed in two separate sessions to ensure that mechanical measurements would not interfere with the metabolic acquisitions. Foot and ankle markers were placed also in the energetics session, and the correspondence of spatiotemporal parameters across the two sessions was checked.

### Locomotion efficiency

Locomotion efficiency was calculated in two ways. First, as commonly used (e.g., Minetti et al. 1993):

7 $$\mathrm{Efficiency}=\frac{{W}_{\mathrm{TOT}}}{C}.$$

Alternatively, when also considering sliding work:

8 $${\mathrm{Efficiency}}_{\mathrm{WS}}=\frac{{W}_{\mathrm{TOT},\mathrm{ WS}}}{C}.$$

### Statistics

Metabolic cost, mechanical work, recovery, and efficiency were regressed on walking speed. For each case, a first-, second- and third-order model were fitted: the Akaike information criterion was calculated and the model with the lowest score was selected. Heart rate was regressed on $\dot{V}{\text{O}}_{{2}}$ with a mixed-effect model with random intercepts, and participants as random factor. Joint angle time series were normalized into 101 nodes and compared between conditions by one-dimensional statistical parametric mapping (SPM) independent samples *t* tests (Pataky 2012; Pataky et al. 2013). Due to the lack of previous data on walking inside the Omni, no a priori sample size estimation was performed, and the angular comparisons should be considered exploratory (Robinson et al. 2021; Luciano et al. 2021). Analyses were performed using Python 2.7.15 (Van Rossum and Drake 2009), Numpy (Harris et al. 2020), Pandas (McKinney 2010), Spm1d (Pataky 2012), GraphPad Prism 9.2 (La Jolla, CA, USA), and R 3.6.2 (R Core Team 2021) with lme4 (Bates et al. 2015).

## Results

### Metabolic cost

**Fig. 2:**
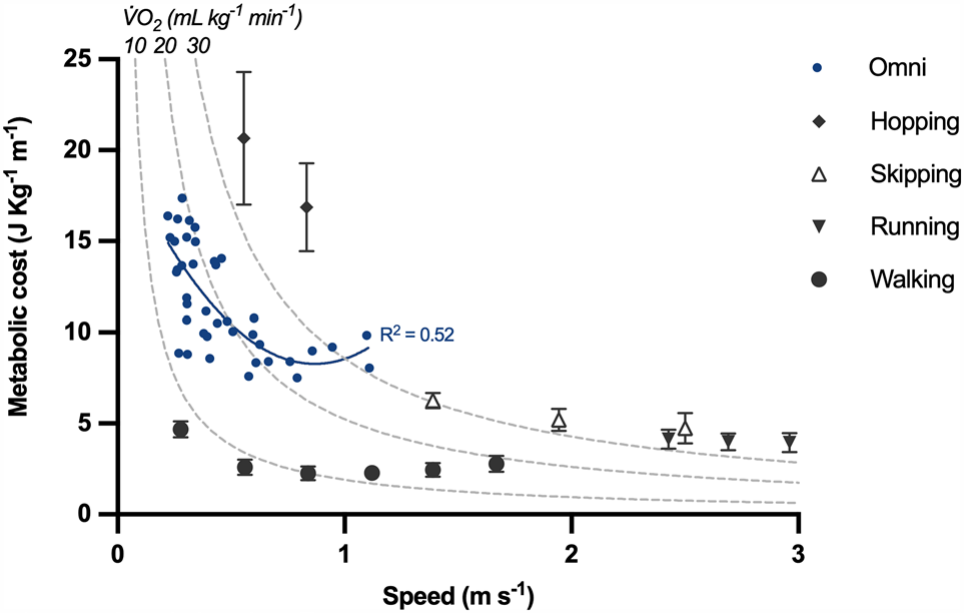
Metabolic cost. The metabolic cost of Omni walking and normal walking, running, skipping, and hopping is shown as a function of speed. Error bars: standard deviation. Solid blue line: second-order fit in the form *y* = 20.2−27.5*x* + 15.8*x*^2^. Dashed hyperbolas: iso-power curves at 10, 20, and 30 mL kg^−1^ min^−1^ of gross oxygen consumption. Data for normal walking from Pavei et al. (2015), for running from Ardigò et al. (1995), for skipping from Minetti et al. (2012), and for hopping from Pavei and Minetti (2016)

**Fig. 3:**
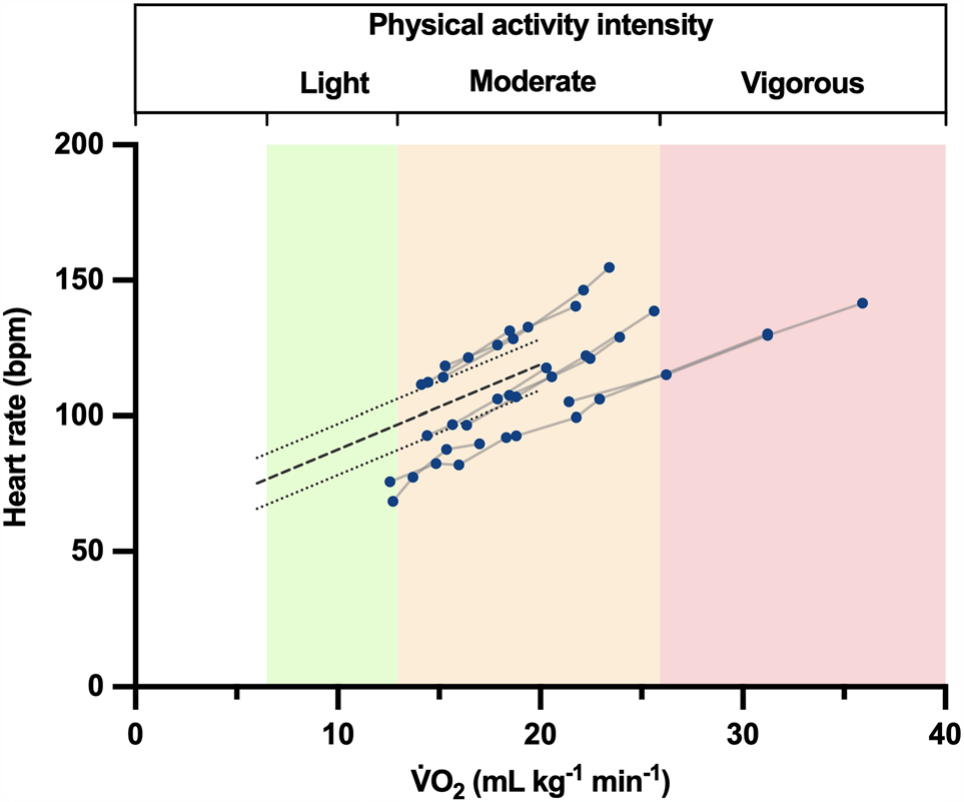
Heart rate and metabolic power for Omni walking. Heart rate is plotted as a function of gross $\dot{V}{\text{O}}_{{2}}$; solid gray lines connect individual participant observations. The dashed black thick line shows the fixed effect for normal walking (data from Pavei et al. 2015), with dotted lines showing the boundaries for the 95% CI. Physical activity intensity is plotted based on the average MET (light: MET < 1.5; moderate: 3 < MET < 6; vigorous: MET > 6; World Health Organization 2020)

The metabolic cost of Omni walking was higher than that of normal walking, running, and skipping, but lower than that of hopping (Fig. 2; Supplementary Fig. 1), and decreased with speed. The relation between C and speed was best fit by a second-order polynomial in the form *y* = 20.2−27.5*x* + 15.8*x*^2^ (*R*^2^ = 0.52; p_B1_ = 0.0004, p_B2_ = 0.0070; Supplementary Data 2), with a minimum at ~ 0.9 m s^−1^. As shown by the iso-metabolic power curves in Fig. 2, walking in the Omni required a metabolic rate ranging from ~ 10 to ~ 30 mLO_2_ kg^−1^ min^−1^, which corresponds to an average MET of 4.6 ± 1.4 (mean ± standard deviation; range: 2.3 to 9.1). Heart rate increased linearly with $\dot{V}{\text{O}}_{{2}}$ (Fig. 3, Supplementary Table 1): the mixed-effect model found a fixed effect of $\dot{V}{\text{O}}_{{2}}$ on heart rate of 3.5 [3.1; 3.8] (*p* < 0.0001) for Omni walking, and 3.1 [2.8; 3.5] for normal walking (*p* < 0.0001; Supplementary Tables 1 and 2).

### Mechanical work

**Fig. 4:**
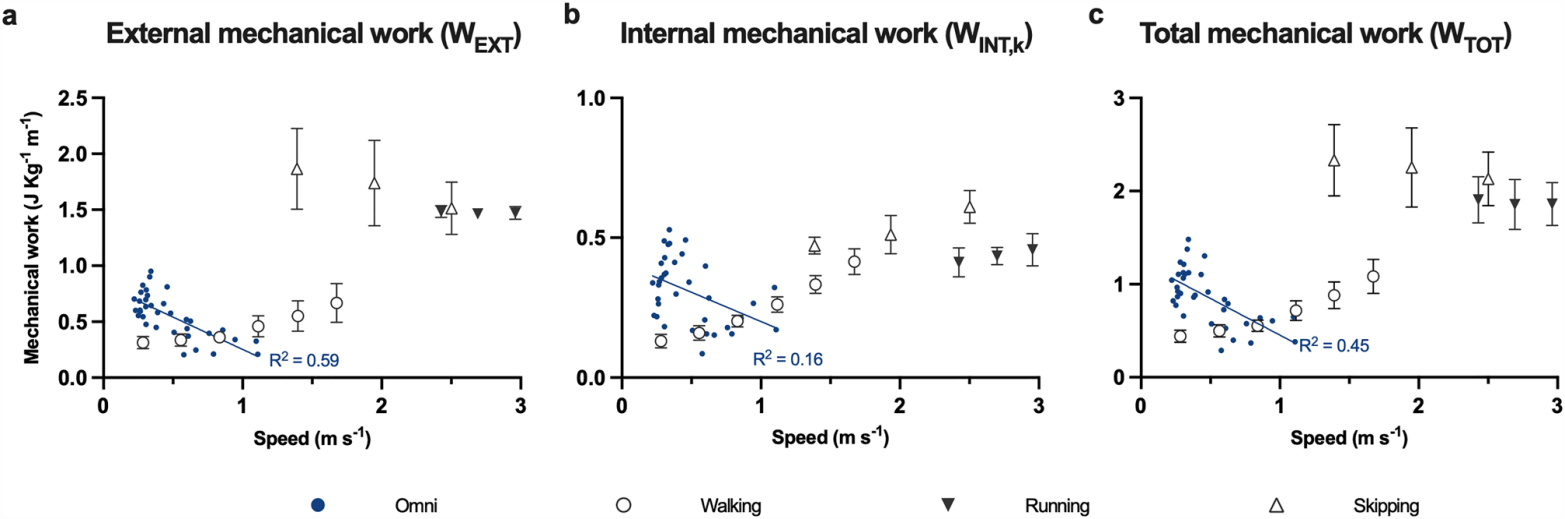
Mechanical work. External, internal, and total mechanical work are shown as a function of speed. Solid blue lines: first-order fits in the forms **a** *y* = 0.83−0.58*x* (*R*^2^ = 0.53), **b** *y* = 0.41−0.21*x* (*R*^2^ = 0.19), and **c** *y* = 1.24−0.79*x* (*R*^2^ = 0.43). Error bars: standard deviation. Sources for other gaits: see the legend of Fig. 2. To make data visualization clearer, hopping data are omitted since their values are outside the axis range, but they are reported in the Supplementary Fig. 2; the range of vertical axes differs among plots

**Fig. 5:**
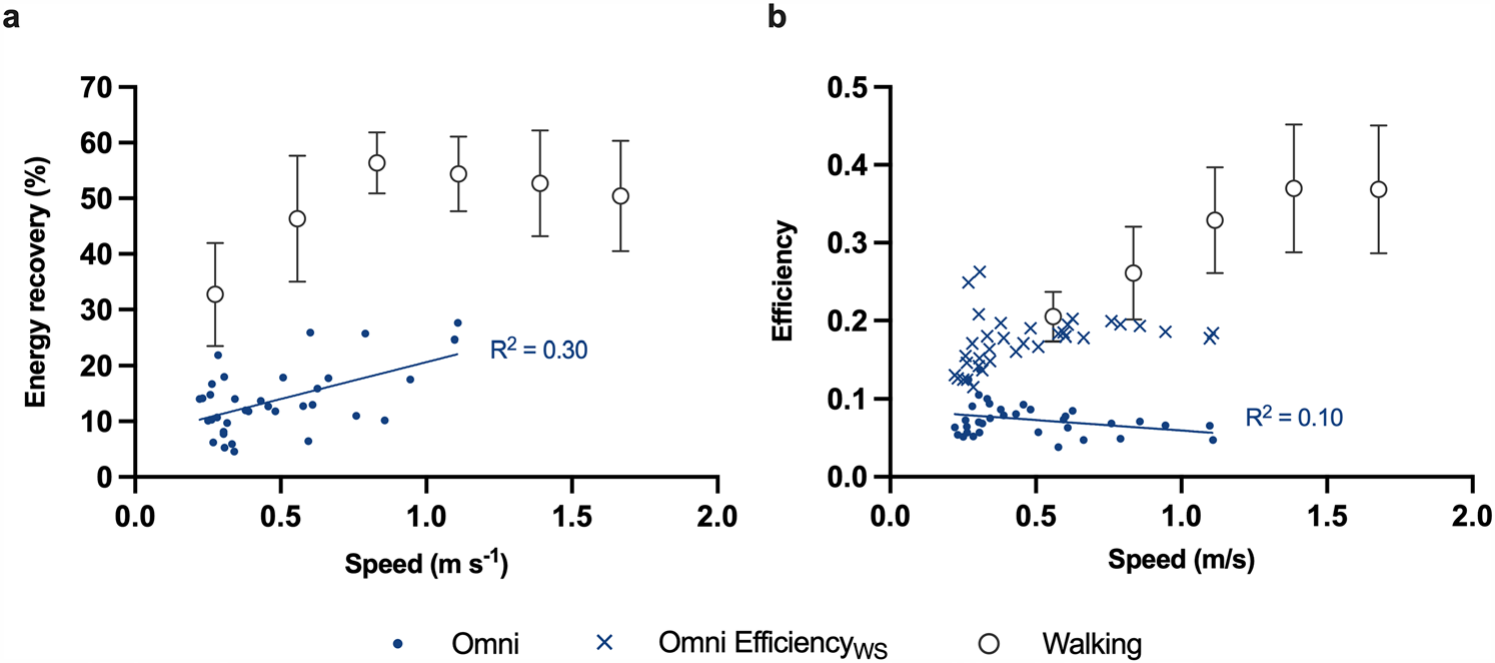
Energy recovery and efficiency. **a** Energy recovery for Omni walking and normal walking is plotted as a function of speed. Solid blue line: first-order fit in the form *y* = 7.39 + 13.26*x* (*R*^2^ = 0.30). **b** Efficiency of Omni walking and normal walking is plotted as a function of speed. Cross signs (Efficiency_WS_) represent the Omni walking efficiency when also the sliding work is considered (see Eq. 5). Solid blue line: first-order fit for efficiency in the form *y* = 0.09−0.03*x* (*R*^2^ = 0.10). Error bars: standard deviation. Data for normal walking from Pavei et al. (2015)

W_EXT_, W_INT,k_, and W_TOT_ of Omni walking decreased linearly with speed (Fig. 4), with best-fit relations of *y* = 0.83−0.58*x* (*R*^2^ = 0.53; *p* < 0.0001), *y* = 0.41−0.21*x* (*R*^2^ = 0.19; *p* = 0.0086), and *y* = 1.24−0.79*x* (*R*^2^ = 0.43; *p* < 0.0001), respectively. Mechanical work was higher for Omni walking compared to normal walking at lower speeds, but such a difference was not evident at higher speeds (Fig. 4). Energy recovery increased with speed for Omni walking (*y* = 7.39 + 13.26*x*; *R*^2^ = 0.30; *p* = 0.0007) and was always lower than normal walking (Fig. 5a). Walking efficiency was nearly constant with speed in Omni, with a best-fit relationship of *y* = 0.09−0.03*x* (*R*^2^ = 0.10, *p* = 0.0710), and lower than normal walking (Fig. 5b). However, when also the mechanical work against sliding frictions was considered, the efficiency of Omni walking was close to that of normal walking at similar speeds (Fig. 5b). Supplementary Data 2 shows all the regression results and the 95% confidence intervals for the fit parameters.

### Kinematics

**Fig. 6:**
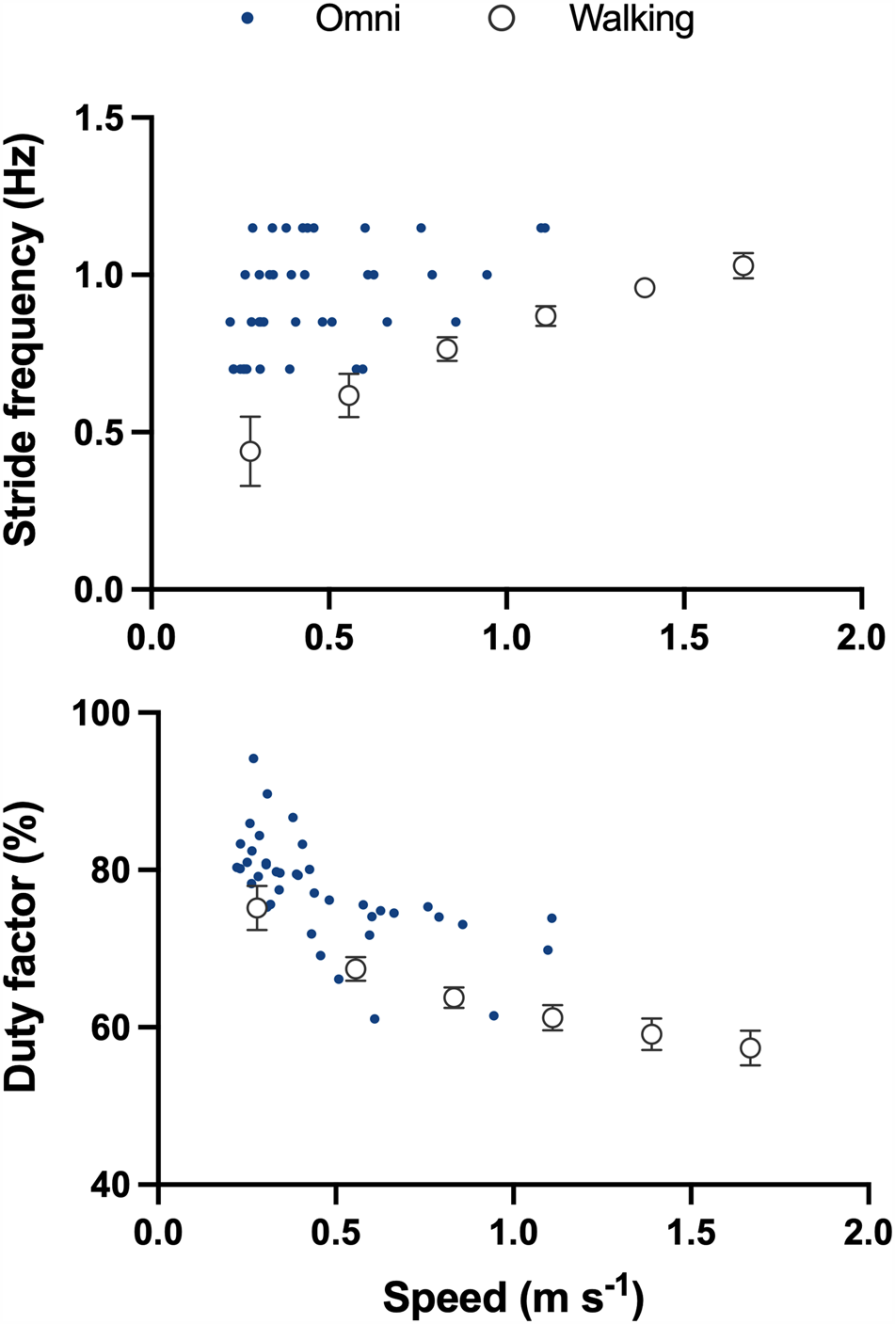
Spatiotemporal parameters. Stride frequency and duty factor for Omni walking (blue dots) and normal walking (empty dots) are plotted as a function of speed. Data for normal walking from Pavei et al. (2015)

**Fig. 7:**
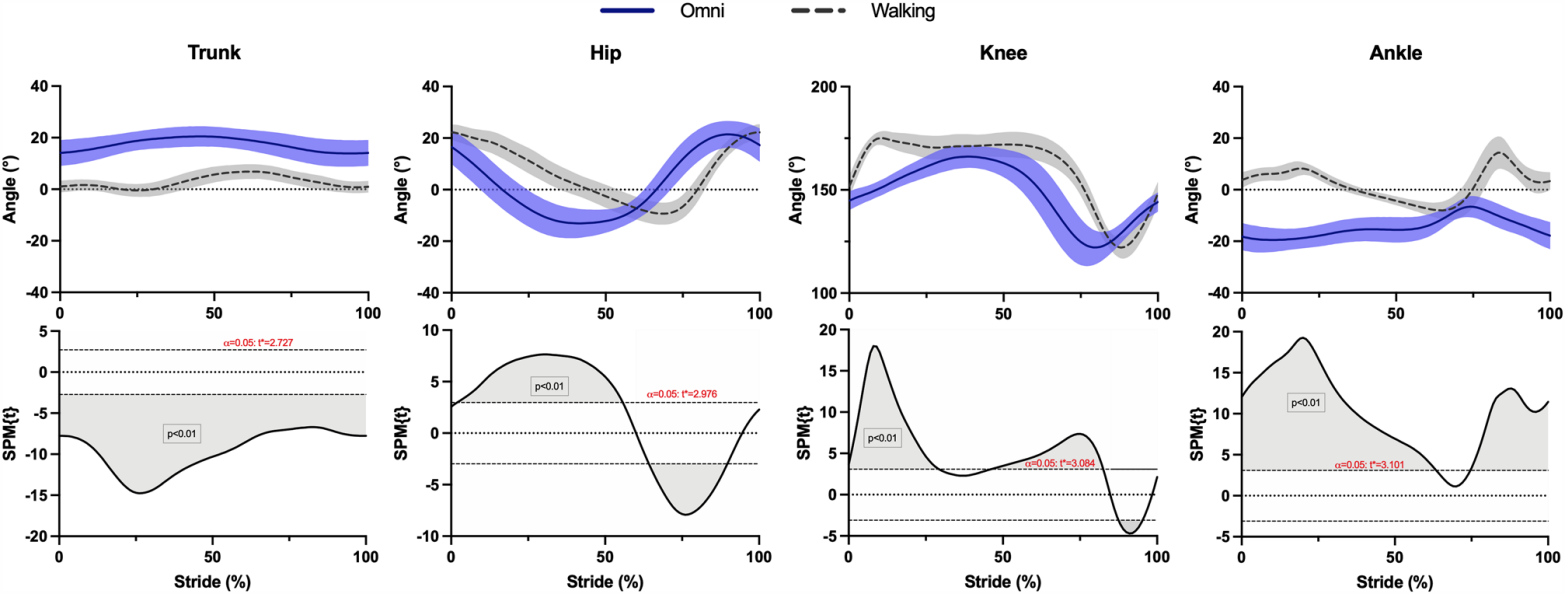
Angular kinematics. Sagittal projections of trunk, hip, knee, and ankle joint angles during Omni walking and normal walking at 0.56 m s^−1^. Shaded blue and gray areas: standard deviation for Omni and normal walking, respectively. Data for normal walking from Pavei et al. (2015)

During Omni walking, increasing the imposed stride frequency increased speed and decreased double contact time, while stride length and duty factor remained almost unchanged (Supplementary Fig. 3). Compared to normal walking, Omni walking had a narrower speed range, higher stride frequency and duty factor at lower speeds, and less evident differences in spatiotemporal parameters at higher speeds (Fig. 6). The trunk was consistently more forward leaned and the ankle more dorsally flexed; the hip was more extended and the knee more flexed in the first half of the stride during Omni walking, whereas both hip and knee were more flexed during the second half (Fig. 7; Supplementary Figs. 4 and 5).

## Discussion

The metabolic cost of Omni walking was higher than that of normal walking and running—the two most common human gaits—as well as higher than that of skipping, a seldom adopted, highly demanding asymmetric gait. The relationship between C and speed for Omni walking was also *U*-shaped; however, the minimum of C occurred at a slower speed than for normal walking (Fig. 2). These increased energy demands have implications for the use of multidirectional treadmills in physical activity promotion and rehabilitation. For example, 1 h of active video gaming at a comfortable speed of 0.5 m s^−1^ would result in a 70 kg player burning 300 kcal above resting values; the same number of calories would be burned when walking for 90 min at 1.4 m s^−1^ or running for 27 min at 2.8 m s^−1^. The mean MET was 4.6 ± 1.4, which classifies Omni walking as a moderate-intensity physical activity according to the World Health Organization guidelines (World Health Organization 2020; Bull et al. 2020). Compared to normal walking, this higher intensity could limit the use of walking on multidirectional treadmills for gait rehabilitation in patients for whom such exercise intensity may be too high. Indeed, the metabolic power for Omni walking ranged from 10 to over 30 mlO_2_ kg^−1^ min^−1^, which may be a high fraction of the maximal aerobic power for untrained individuals and those with conditions impairing their exercise capacity (Fitzgerald et al. 1997; Schneider 2013; Ried-Larsen et al. 2017; Sietsema et al. 2020): this may shorten the amount of time during which such people could walk on multidirectional treadmills. For a comparison, normal walking at self-selected speed requires about 10 mLO_2_ min^−1^ kg^−1^ (3 METs), with a minimum of 5 mLO_2_ min^−1^ kg^−1^ (1.4 METs) at 1.3 m s^−1^ and downhill slope of −18% (Ardigò et al. 2003). On the other hand, the higher metabolic power of walking on multidirectional treadmills could make such activity beneficial to improve cardiorespiratory fitness and the effects of an inactive and sedentary lifestyle when included in physical activity promotion programs and high-intensity interval training interventions (World Health Organization 2020; MacInnis and Gibala 2017; Ekelund et al. 2016). Of note, even though Omni walking has a higher metabolic cost than running and skipping, this does not mean that exercise intensity is higher as well: as shown by the iso-power curves in Fig. 2, running and skipping generally have a higher MET since they are performed at a higher progression speed. The metabolic power required for Omni walking was also strongly correlated with heart rate, with a similar bpm: $\dot{V}{\text{O}}_{{2}}$ relation to that of normal walking and large between-participant variability in the regression intercepts. These results suggest that heart rate can be used to monitor exercise intensity during walking on multidirectional treadmills (e.g., as done by Dębska et al. 2019), provided that individual differences in resting heart rate are taken into account.

Differences in metabolic demands across and within gaits can often be attributed to differences in mechanical work, since this is one of the main determinants of the metabolic cost (for a review, see Peyré-Tartaruga et al. 2021). In this study we used the same approach by calculating the ‘traditional’ sources of mechanical work when walking on the multidirectional treadmill; however, differences in W_TOT_ alone did not fully explain the differences in C. Indeed, *W*_TOT_ decreased linearly with speed, whereas C followed a curvilinear trend. Moreover, the efficiency of Omni walking was consistently lower than that of normal walking: it would have been the same if W_TOT_ had explained all the increase in C. Analyzing the components of W_TOT_ can shed further light on the mechanisms underlying its variations between gaits. External mechanical work decreased with speed for Omni walking while it increased for normal walking, with an increasing energy recovery for both gaits. As W_EXT_ can also be considered as (1−Recovery)(*W*_forward_ + *W*_vertical_) (Luciano et al. 2022), where (*W*_forward_ + *W*_vertical_) is the sum of the increases in the kinetic energy of the forward motion and the vertical energy of BCoM, respectively, such opposite speed-dependency for W_EXT_ is due to a divergent trend for this last term, rather than for the ability to behave in a pendulum-like manner. On the other hand, differences in W_INT,k_ can be explained by differences in limb kinematics. At a given speed, W_INT,k_ is proportional to *f*(1 + (*d*/(*1*−*d*))^2^) (Minetti and Saibene 1992; Minetti 1998), where *f* is the stride frequency and *d* is the duty factor: at lower speeds, stride frequency and duty factor were higher for Omni walking than normal walking, but at higher speeds the differences between the two activities tended to disappear (Fig. 6). As a result, W_INT,k_ was higher in multidirectional treadmill walking only at speeds below 1 m s^−1^. Of note, in this study we compare Omni walking at imposed frequencies with reference data on treadmill walking at fixed speeds. However, observed differences in cost, mechanical work, and kinematics are largely greater than those attributable to inter-participant variability and variations in stride frequencies (Minetti et al. 1995; Umberger and Martin 2007; Stoquart et al. 2008; Pavei et al. 2015).

While the work against external frictions is negligible during normal walking, it may be a major determinant of the high metabolic cost of walking on a multidirectional treadmill. Indeed, apparent differences in efficiency between the two gaits were largely reduced when the work done against sliding friction was taken into account. Some further factors were not included in the present analysis, but may contribute to the high cost of walking on the multidirectional treadmill: among them, internal frictional mechanical work (Minetti et al. 2020) done within joints. Such term was not included in W_TOT_ since its partitioning with W_INT,k_ has not been solved yet, and little can be inferred about differences in internal frictional mechanical work between Omni and normal walking. Additionally, the denominator of efficiency is given by metabolic cost, which also includes the energy spent by muscles that do not perform mechanical work, such as those contracting isometrically or co-contracting. When walking on a multidirectional treadmill, a greater fraction of active muscles may be activated to comply with stability and postural needs due to the more crouched posture—as hinted also by the higher electromyographic activity of the lower limbs reported by Soni and Lamontagne (2020)—without generating mechanical work. Finally, during normal walking, some energy is saved due to storage and release of elastic energy by the tendons and connective structures of the hip and ankle joints (Fukunaga et al. 2001; Eng et al. 2015) and due to optimized muscle and tendon gearing of knee extensors and plantar flexors (Monte et al. 2022); however, the different kinematics of such joints during Omni walking may hamper these energy-saving mechanisms.

The unique kinematic patterns, BCoM motion, and limb mechanics of walking on multidirectional treadmills distinguish it from normal walking and highlight the need for caution when evaluating its use in gait rehabilitation. Compared with normal walking, Omni walking showed a more flexed trunk, a dorsally flexed ankle, and a different angular displacement at the hip and ankle joints. Moreover, participants attained slower speeds than in normal walking and with different spatiotemporal parameters, as previously observed by other authors (Jochymczyk-Woźniak et al. 2019; Soni and Lamontagne 2020). Together with differences in mechanical work and metabolic cost, such observations challenge the ability of multidirectional treadmills and virtual reality emulators to mimic normal walking. Further studies should test whether observed features of walking on the Omni treadmill can be generalized to other multidirectional treadmills. We can speculate, however, that this should be the case for at least some of them. For instance, also Soni and Lamontagne (2020) found that, compared to normal walking, people had a lower stride length when walking on a different multidirectional treadmill. Moreover, all passive treadmills require to perform work against sliding frictions, and the coefficients of these frictions should be maintained within a narrow range to prevent surfaces from becoming unrealistically slippery.

## Conclusions

Walking on a multidirectional treadmill has a higher metabolic cost and different mechanics and kinematics than normal walking. These findings question the use of multidirectional treadmills in rehabilitation when the goal is to mimic normal gait, but support their use to promote high-intensity exercise and physical activity in the general population.

### Supplementary Information

Below is the link to the electronic supplementary material.

Supplementary file1 (XLSX 43 KB)

Supplementary file2 (XLSX 11 KB)

Supplementary file3 (DOCX 383 KB)

Supplementary Video 1 (MOV 18545 KB)

## Data Availability

Individual-level metabolic, mechanical, and kinematic data can be found in Supplementary Data 1.

## Abbreviations

C: Metabolic cost (J kg^−^^1^ m^−^^1^)
BCoM: Body center of mass
EqO_2_: Energy equivalent of oxygen (J mL^−^^1^)
KE: Kinetic energy (J)
MET: Metabolic equivalent of task (dimensionless)
PE: Potential energy (J)
TE: Total mechanical energy (J)
W_EXT_: External mechanical work (J kg^−^^1^ m^−^^1^)
*W*_INT,k_: Internal kinetic mechanical work (J kg^−^^1^ m^−^^1^)
W_slide_: Work done against sliding frictions (J kg^−^^1^ m^−^^1^)
W_TOT_: Total mechanical work (J kg^−^^1^ m^−^^1^)
$\dot{V}{\text{O}}_{{2rest}}$: Oxygen consumption at rest (mL min^−^^1^ kg^−^^1^)
$\dot{V}{\text{O}}_{{2ss}}$: Oxygen consumption at steady state (mL min^−^^1^ kg^−^^1^)
