# Supplementary material for: Metabolic cost and mechanical work of walking in a virtual reality emulator
Source: Eur J Appl Physiol. 2023 Sep 30;124(3):783–92. doi: 10.1007/s00421-023-05306-0 (PMC10879236; doi:10.1007/s00421-023-05306-0)
Supplement: Supplementary file 3 — Supplementary file3 (DOCX 383 KB) [file 421_2023_5306_MOESM3_ESM.docx]

**Metabolic cost and mechanical work of walking in a virtual reality emulator**

*Francesco Luciano*, Alberto E. Minetti, Gaspare Pavei*

Locomotion Physiomechanics Laboratory – Division of Physiology

Department of Pathophysiology and Transplantation, University of Milan, Italy

** Corresponding author.*

*Via Mangiagalli 32, 20133 Milano, Italy.
E-mail address: francesco.luciano1@unimi.it*

**SUPPLEMENTARY METHODS**

**S1. Calculation of the sliding friction coefficient between shoes and treadmill**

In order to calculate static sliding friction coefficient (μ_s_), a shoe was loaded with a 20-kilogram mass and placed on the flat, central portion of the Omni platform. The entire platform was then tilted until the shoe spontaneously started sliding due to gravity acceleration. Under this condition, the modulus of the tangent projection of the weight of the loaded shoe (p_//_) equals that of the sliding friction force acting on it (F_f_):

$$p_{//}=F_{f}$$

And given α the tilting angle of the platform:

$$p\sin\left( \alpha\right)=p\cos\left( \alpha\right)\mu_{s}$$

$$\mu_{s}=tan(\alpha)$$

Similarly, the dynamic sliding friction coefficient (μ_d_) was calculated as the tangent of the angle at which the shoes could continue sliding after being externally accelerated.

**SUPPLEMENTARY FIGURES AND TABLES**

*Supplementary Figure 1*. *Metabolic cost of Omni walking as a function of speed.* Each colour represents an individual participant.

*Supplementary Figure 2.*  *Mechanical work as a function of speed.* Solid red lines: first-order fits in the forms (a) y = 0.83 -0.58x (R^2^=0.53), (b) y = 0.41 -0.21x (R^2^=0.19), and (c) y = 1.24 -0.79x (R^2^=0.43). Error bars: standard deviation. Data for normal walking from Pavei et al. (2015), for running from Ardigò et al. (1995), for skipping from Minetti et al. (2012), for hopping from Pavei and Minetti (2016). The range of vertical axes differs among plots.

*Supplementary Figure 3*. *Kinematics.* Stride length, speed, double contact time, and duty factor during walking Omni walking are plotted as a function of the imposed stride frequency. Grey lines connect individual participant observations.

*Supplementary Figure 4. Angular kinematics.* Sagittal projections of trunk, hip, knee, and ankle joint angles in Omni walking and normal walking at 0.28 m s^-1^. Shaded blue and grey areas: standard deviation. Data for normal walking from Pavei et al. (2015).

*Supplementary Figure 5.* *Effect of speed on angular kinematics of Omni walking.* Shaded green and blue areas: standard deviation.

*Supplementary Table 1.* *Heart rate and metabolic power for Omni walking.* Results of the mixed effect model regressing heart rate over V̇O_2_, with participants as random factors.

| *Fixed effects* | | |
| --- | --- | --- |
|  | *Estimate [95% CI]* | *t-value* |
| *(Intercept)* | 42.7 [30.9; 56.3] | 6.8 |
| V̇O_2_ | 3.5 [3.1; 3.8] | 19.2 |
|  | | |
| *Random effects [95% CI]* | | |
|  | *Variance* | *St. dev* |
| *Participant* | 282.6 | 16.8 |
| *Residual* | 12.9 | 3.6 |

*Supplementary Table 2.* *Heart rate and metabolic power for normal walking.* Results of the mixed effect model regressing heart rate over V̇O_2_, with participants as random factors. Data from Pavei et al. (2015)

| *Fixed effects* | | |
| --- | --- | --- |
|  | *Estimate [95% CI]* | *t-value* |
| *(Intercept)* | 56.4 [47.0; 65.8] | 12.04 |
| V̇O_2_ | 3.1 [2.8; 3.5] | 18.7 |
|  | | |
| *Random effects [95% CI]* | | |
|  | *Variance* | *St. dev* |
| *Participant* | 218.3 | 14.8 |
| *Residual* | 14.5 | 3.8 |

**References**

Ardigò LP, Lafortuna C, Minetti AE, et al (1995) Metabolic and mechanical aspects of foot landing type, forefoot and rearfoot strike, in human running. Acta Physiol Scand 155:17–22. https://doi.org/10.1111/j.1748-1716.1995.tb09943.x

Minetti AE, Pavei G, Biancardi CM (2012) The energetics and mechanics of level and gradient skipping: Preliminary results for a potential gait of choice in low gravity environments. Planetary and Space Science 74:142–145. https://doi.org/10.1016/j.pss.2012.06.004

Pavei G, Biancardi CM, Minetti AE (2015) Skipping vs. running as the bipedal gait of choice in hypogravity. J Appl Physiol (1985) 119:93–100. https://doi.org/10.1152/japplphysiol.01021.2014

Pavei G, Minetti AE (2016) Hopping locomotion at different gravity: metabolism and mechanics in humans. J Appl Physiol (1985) 120:1223–1229. https://doi.org/10.1152/japplphysiol.00839.2015
